# A Randomized, Double-Blind, Placebo-Controlled, Cross-Over Study to Evaluate the Efficacy of Aqualief^TM^ Mucoadhesive Tablets in Head and Neck Cancer Patients Who Developed Radiation-Induced Xerostomia

**DOI:** 10.3390/cancers13143456

**Published:** 2021-07-10

**Authors:** Nicola Alessandro Iacovelli, Rossana Ingargiola, Nadia Facchinetti, Marzia Franceschini, Domenico Attilio Romanello, Paolo Bossi, Cristiana Bergamini, Salvatore Alfieri, Stefano Cavalieri, Giovanna Baron, Giancarlo Aldini, Laura Locati, Ester Orlandi

**Affiliations:** 1 Radiation Oncology Unit 2, Fondazione IRCCS Istituto Nazionale dei Tumori di Milano, Via Venezian 1, 20133 Milan, Italy; rossana.ingargiola@cnao.it (R.I.); nadia.facchinetti@cnao.it (N.F.); marzia.franceschini@istitutotumori.mi.it (M.F.); domenico.romanello@cnao.it (D.A.R.); ester.orlandi@cnao.it (E.O.); 2 Head and Neck Medical Oncology, Fondazione IRCCS Istituto Nazionale dei Tumori di Milano, Via Venezian 1, 20133 Milan, Italy; paolo.bossi@unibs.it (P.B.); cristiana.bergamini@istitutotumori.mi.it (C.B.); salvatore.alfieri@cro.it (S.A.); stefano.cavalieri@istitutotumori.mi.it (S.C.); laura.locati@istitutotumori.mi.it (L.L.); 3 Department of Pharmaceutical Sciences (DISFARM), Università degli Studi di Milano, Via Mangiagalli 25, 20133 Milan, Italy; giovanna.baron@unimi.it (G.B.); giancarlo.aldini@unimi.it (G.A.); 4 Radiation Oncology Unit 1, Fondazione IRCCS Istituto Nazionale dei Tumori di Milano, Via Venezian 1, 20133 Milan, Italy

**Keywords:** Aqualief^TM^, xerostomia, radiotherapy, head and neck cancer, carnosine, karkadé

## Abstract

**Simple Summary:**

Xerostomia, the subjective complaint of dry mouth, is caused by therapeutic interventions or diseases. Nowadays, radiotherapy in patients with head and neck cancer (HNC) stands out as one of the most important causes of xerostomia. Currently available therapies for the treatment of xerostomia are still less than optimal and xerostomia still represents an unmet clinical need. In this article, we present the results of a clinical study with a new product, Aqualief^TM^, in patients treated with curative radiotherapy for HNC. The results show that Aqualief^TM^ stimulated salivation in these patients and reduced the pH drop that was observed in an equivalent population of patients treated with placebo. Moreover, no serious, treatment-related adverse events were observed. These encouraging results suggest that Aqualief^TM^ may become a promising tool for the treatment of radiotherapy-related xerostomia. In addition, the results also suggest that Aqualief^TM^ may have positive effects in the maintenance of oral health.

**Abstract:**

Xerostomia, the subjective complaint of dry mouth, is caused by therapeutic interventions or diseases. Nowadays, radiotherapy (RT) in patients with head and neck cancer (HNC) stands out as one of the most important causes of xerostomia. Currently available therapies for the treatment of xerostomia are still less than optimal and xerostomia still represents an unmet clinical need. In this article, we present the results of a prospective clinical study with a new product, Aqualief^TM^, in patients treated with curative RT with or without chemotherapy for HNC. Aqualief^TM^ is based on two main ingredients, carnosine and karkadé, which have acid buffering and antioxidant properties. The study was performed on 30 patients, with 4 of the patients being lost during the study period. Each patient received randomly one of the two treatments, Aqualief^TM^ or placebo, for 8 days. After a 10-day wash-out period, each patient received the other treatment for a further 8 days. The results show that Aqualief^TM^ stimulated salivation in these patients and reduced the pH drop that was observed in an equivalent placebo-treated population of patients. Moreover, no serious, treatment-related adverse events were observed. Aqualief^TM^ has shown positive results, although with limitations due to unsuccessful trial accrual. Therefore, it may be further investigated as a tool for the treatment of RT-related xerostomia.

## 1. Introduction

Xerostomia, defined as the subjective complaint of dry mouth, is one of the most prevalent and challenging adverse effects for head and neck cancer (HNC) patients treated with radiotherapy (RT) in definitive or adjuvant setting with or without concomitant chemotherapy (CHT). It represents a toxicity that can resolve over time [1,2,3], but often translates into a permanent condition that seriously affects swallowing, speaking and oral health, impairing several domains of patients’ quality of life (QoL) [4,5]. Symptomatically, xerostomia may range from mild discomfort to severe oral disease accompanied by signs and symptoms affecting the oral cavity, including mucous membranes, lips, tongue, salivary glands and teeth [6,7]. In the most severe cases it can cause severe depression [8].

Although radiation-induced xerostomia (RIX) is multifactorial, it is primarily the consequence of damage to the major and minor salivary glands that are usually included in the radiation fields or are in their close proximity [9,10]. Thus, the severity of glandular injury and potential for recovery depends on the irradiated gland volume, the cumulative radiation dose and the capability of surviving cells to repopulate [11]. Such injury causes diminution in function of the salivary glands and the consequences are reduction in saliva volume, consistency, pH, immunoglobulins and antimicrobial proteins [12,13,14].

The advent of highly conformal RT, such as intensity-modulated radiation therapy (IMRT) and volumetric modulated arc therapy (VMAT), has greatly reduced the rate of late RIX in HNC patients, thereby ameliorating their QoL [15,16]. In particular, a meta-analysis of five studies including a total of 871 HNC patients reported a significant overall benefit in favor of IMRT (hazard ratio—HR = 0.76; 95% CI: 0.66, 0.87; *p* < 0.0001) as regards xerostomia scores grade 2–4, with similar loco-regional control and overall survival [16]. Although the first step in management of RIX, is to optimize IMRT in order to preserve salivary gland function in HNC patients without compromising target coverage, medical interventions and measures can play a role in further limiting injury to the salivary glands and, consequently, reducing the risk of xerostomia.

Overall, two main clinical approaches are used to manage RIX. First, compounds that act by stimulating the residual secretory capacity of the salivary glands [17,18,19]. Three reviews, one of which included a meta-analysis, reported that sialagogue medications (such as pilocarpine and cevimeline) are appropriate for patients with some degree of residual salivary gland functional parenchyma [14,20,21]. Other treatment modalities could not be recommended on the basis of current evidence. Second, use of saliva substitutes in order to reduce and palliate xerostomia symptoms [14,21,22]. This latter approach is actually the only one that can be pursued when salivary glands can no longer be stimulated efficiently, but the efficacy of available substitutes is dubious [14,23]. Overall, the therapeutic armamentarium currently available appears to be still unsatisfactory and xerostomia represents an important unmet clinical need.

Aqualief^TM^ is based on two main ingredients, carnosine and dried calyces of Hibiscus sabdariffa (karkadé). These two ingredients are mixed in defined proportions in order to maintain a stable pH of the oral cavity and to improve the acid buffering potential of the saliva. Carnosine was included in the preparation because of its anti-inflammatory, antioxidant, antiglycation and chelating effects [24]. Karkadé, on the other hand, has been reported having antioxidant effects, broad spectrum antimicrobial activity and anti-inflammatory effects [25]. Aqualief^TM^ is designed as a mucoadhesive tablet that adheres to the internal side of the cheek so to afford a prolonged action. It is expected to gradually release the ingredients over a period of at least two hours. Aqualief^TM^ has been recently tested in an Italian double-blind trial in 70 patients with grade 1–2 xerostomia from a wide spectrum of diseases [26]. In particular, Aqualief^TM^ was reported to be effective in regulating the saliva pH, in increasing saliva production and improving dry mouth symptoms in xerostomic patients [26].

Subsequently, we designed a randomized, double-blind, placebo-controlled, cross-over trial aimed at evaluating the effectiveness of Aqualief^TM^ compared to placebo in treating RIX in an HNC patients cohort treated with curative IMRT. Herein, we report the results of this study.

## 2. Materials and Methods

### 2.1. Study Design

The study was conducted according to the guidelines of the Declaration of Helsinki and approved by the Institutional Ethics Committee of Fondazione IRCCS Istituto Nazionale dei Tumori di Milano (INT 151/17—ClinicalTrials.gov Identifier NCT03601962). All patients provided a written informed consent, which they could withdraw at any time without prejudicing further medical care.

It was a prospective, randomized, double-blind, placebo-controlled, cross-over study.

Eligible patients were randomized in a 1:1 ratio and treated for 8 days with Aqualief^TM^ (400 mg oral mucoadhesive tablet per day), versus placebo (phase 1) and then, following a 10-day wash-out period, for further 8 days with placebo versus Aqualief^TM^ (phase 2). In this phase, the study was designed as a double-blind, cross-over study and neither the patient nor the clinical site personnel knew which treatment was being administered during both phases of the study. The identity of the treatments could not be revealed except in an emergency under the discretion of the Investigator. The Principal Investigator received a study treatment identification key in the form of sealed envelopes containing the kit number and the corresponding treatment. The patient randomization list was generated using the module RALLOC of STATA/IC 12.1 for Windows (Stata Corp LP, College Station, TX, USA). Once eligibility was established the Investigator assigned the first available number of study treatment. Kits were identified by a numeric code, 001, 002, 003, etc. and by letter A and B. In the first double blind phase the Investigator should assign the lower available kit number, letter A and in the second double blind phase the same kit number, letter B. The assigned number and letter were recorded by the Investigator.

Exposure to Aqualief^TM^ was continued on a voluntary basis as an open phase which lasted another twelve weeks (phase 3). Visits were performed before the beginning of each study phase (baseline) and at the end of each phase (i.e., day 8 of the first phase, day 8 of the second phase and at the end of the open phase).

### 2.2. Study Product

Aqualief^TM^ (currently marketed under the brand name Salifluss^®^, Metis Healthcare srl, Milan, Italy) consists of mucoadhesive tablets with smooth surfaces, each 400 mg. The dissolution time of the tablet is approximately 2 h in normal saliva conditions. As already mentioned, the study product contains two main ingredients mixed in a specific proportion: carnosine and dried calyces of *Hibiscus sabdariffa* L. (karkadé). Placebo tablets lacked the two main ingredients but, otherwise, were the same as Aqualief^TM^. Placebo tablets contained amylopectin; carboxylate (carbomer), sorbitol (D-Glucitol), hydroxypropylcellulose, magnesium stearate FU–Ph EU, aroma and sucralose.

Aqualief^TM^ is reported to the Italian Ministry of Health with ID number 83383. At recommended doses, the intake quantities of the substances meet requirements and limitations set by the Italian Ministry of Health. Due to the presence of flavonoids in Hibiscus Sabdariffa L. medical advice is suggested when used during pregnancy.

Aqualief^TM^ or placebo were taken three times a day for 8 consecutive days, specifically: one tablet in the morning after breakfast and after brushing the teeth; one tablet after lunch around 15:00 h in the afternoon always after brushing the teeth; one tablet in the evening after dinner and after brushing the teeth, at least two hours before bedtime.

The patients were asked to place the tablet in the fornix at the sixth upper molar and allow it to adhere to the cheek and dissolve without chewing or lingual stimulation.

Each patient received randomly one of the two treatments, Aqualief^TM^ or placebo, for 8 days. After a 10-day wash-out period, each patient received the other treatment for a further 8 days.

### 2.3. Patient Selection

Patients eligible for inclusion in this study had to meet all of the following criteria: 18 years of age or older; willing and able to give signed informed consent and, in the opinion of the Investigator, to comply with the protocol tests and procedures; subjects presenting hyposalivation of grade 2 or greater (according to Common Terminology Criteria for Adverse Events scale v 4.0) and an objective unstimulated salivary flow between 0.1 and 0.25 mL/min measured at least 6 months after the end of IMRT; treated with curative IMRT in definitive or postoperative setting, with or without CHT for HNCs (including all subsites); completed IMRT since at least 6 months and free from cancer disease; absence of infections in the oral cavity; and absence of antibiotics and antifungal treatments or any dental procedure in the 10 days before each treatment phase of the study.

Exclusion Criteria included the following: contraindications for the administration of carnosine and hibiscus; known hypersensitivity to the components present in the product; subjects taking products or medications to reduce symptoms of salivary gland hypofunction (pilocarpine, etc.); patients with other underlying conditions that can cause xerostomia; use of experimental drugs within 30 days prior to enrollment or during the study; presence of clinical conditions that may interfere with the study evaluations; and pregnant or lactating women.

### 2.4. Patient Treatment and Follow-Up

All patients received Volumetric-Modulated Arc Therapy (VMAT) with two or three coplanar arcs. In the definitive setting, RT total doses were 70, 60 and 50–54 Gy to high, intermediate and low risk clinical target volume, respectively; in postoperative cases total dose to the corresponding volumes were 66, 56–60 and 54 Gy, respectively. In all cases conventional or moderately accelerated fractionation (1.80–2.15 Gy/die) was used, with an overall treatment time of 6.5–7 weeks. RT planning was previously reported [27,28]. According to histology, stage and pathology reports, patients could receive concomitant platinum-based CHT, as per international guidelines (National Comprehensive Cancer Network, NCCN) and institutional Policies [29].

After RT completion, patients were clinically evaluated at predefined intervals, every 3–6 months for the first 3 years and annually thereafter (NCCN). Radiological restaging with the same method of baseline imaging was performed 3 months after treatment end and then prescribed on a regular basis or when deemed necessary according to patients’ disease status. Adverse events (AEs) were graded according to the CTCAE version 4.0.

### 2.5. Enrollment and Interventions

Patients with a minimum follow-up of at least 6 months were eligible for this study. During the visit they were enrolled and completed baseline Xerostomia Evaluation (XQ-I) questionnaire and MD Anderson Dysphagia Inventory (MDADI) questionnaire.

### 2.6. Measurement of Saliva Production and Saliva pH

Unstimulated saliva was collected every 30s for 5 consecutive min in a tube which was then weighed to estimate the flow of resting saliva.

Saliva production was determined by the spit method at baseline (t = 0) and after 8 days of treatment (t = 1). The patient was instructed to assume the treatment up to the visit day, therefore salivary collection was performed during treatment administration.

### 2.7. Patients’ Compliance

The patients’ compliance to treatment was checked by the study staff by counting the number of tablets returned by the patients at each visit. In case compliance to treatment was lower than 50% in phases 1 and 2 of the study, with 4 consecutive days of missed tablets, the patient was withdrawn from the study. Two consecutive days of totally or partially missed tablets just before evaluation of the endpoints, at visits, was reason for withdrawal from the study.

### 2.8. Study Endpoints

Primary Endpoint:

1. Saliva production without mechanical stimulation: change of saliva produced from baseline to 8 days of treatment.

Secondary Endpoints:

2. Determination of pH of the mouth cavity: change from baseline to 8 days of treatment.
3. Xerostomia evaluation (XQ-I questionnaire) [30]: change from baseline to 8 days of treatment
4. MDADI questionnaire [31,32]: change from baseline to 8 days of treatment.
5. Adherence to the treatment by accountability: total tablets used from baseline to 8 days of treatment.
6. Patient’s global satisfaction: patients’ report on the ease of use and palatability of the product from baseline to 8 days of treatment.

### 2.9. Statistical Analysis

Based on previous unpublished data, assuming an increase in the saliva volume collected in five minutes of 0.33 mL after treatment with placebo and of 0.46 after treatment with Aqualief^TM^ and no period effect, with a common standard deviation of 0.35 and a correlation of 0.5, 79 patients were needed to assess a significant difference at alpha = 0.05 with a power of 90%. To take into account a possible 20% drop-out rate, 100 patients were planned to be enrolled.

Descriptive statistics of all relevant variables was performed. Continuous variables were summarized by the number of patients (N), mean, standard deviation, median, minimum and maximum. Where appropriate, 95% confidence intervals for the target variables were estimated. Categorical variables were summarized by the number (N) and the proportion of patients (%). To compare demographic and baseline characteristics between treatment groups, chi-square or *t*-tests were used for discrete and continuous variables, respectively. Statistical analysis between the two time points (baseline and 8 days) of the same groups (placebo and Aqualief^TM^) was performed by paired *t*-test and between the two groups, unpaired *t*-test. The significance level of statistical tests was set at 0.05. The statistical analysis was performed using SAS 9.4 for Windows (SAS Institute Inc., Cary, NC, USA) and GraphPad Prism version 9.0 (Software, La Jolla, CA, USA).

## 3. Results

### 3.1. Study Population

Enrollment started in March 2018 and ended in December 2019. The study was interrupted because of inadequate accrual rate when 30 patients had been recruited. Therefore, no subgroup analysis was performed. The only covariates used were the values at baseline, the period (first or second period) and the crossover treatment sequence (Placebo-Aqualief^TM^ or Aqualief^TM^-Placebo), when applicable. No interaction was investigated. Missing efficacy and missing safety data were not replaced.

There was no screening failure among study patients. Four patients were lost during the study period and were not included in the analysis. Characteristics and treatment details of the study population are shown in Table 1 and Table 2. Table 1 shows patients who received at least one treatment dose (safety population). Table 2, on the other hand, includes patients who completed both 8-day treatment periods (intention to treat (ITT) population) and who were analyzed for the primary efficacy endpoint (see below). No significant differences were observed between treatment groups at baseline when considering the different study parameters (Table 1 and Table 2).

### 3.2. Primary Endpoints

**Figure 1:**
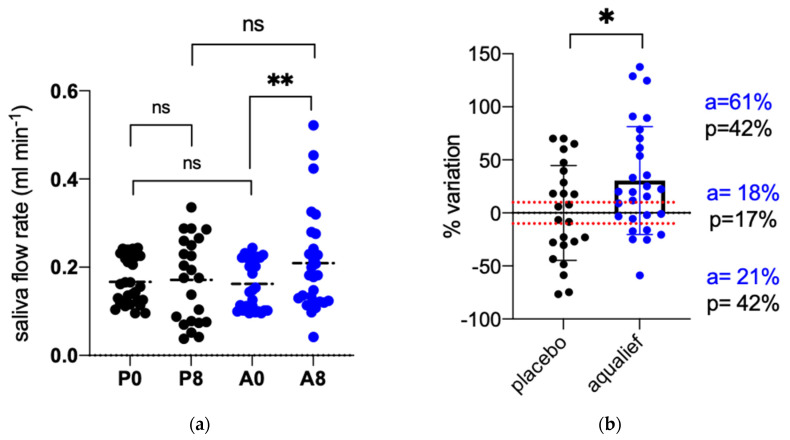
Effect of Placebo and Aqualief^TM^ on salivation. (**a**) Saliva flow rate (mL min^−1^) recorded before (P0,A0) and after 7 days of treatment (P8,A8); (**b**) Data are reported as percent variation of saliva flow rate between the two time points. P, placebo, A, Aqualief^TM^, * *p* < 0.1, ** *p* < 0.01, ns, no significant differences. Statistical analysis between the two time points of the same group was performed by paired *t*-test, between the two groups by unpaired *t*-test.

After one week of treatment with placebo, the saliva flow rate was 0.17 mL min^−1^, not statistically different compared to baseline (0.17 mL min^−1^, *p* = 0.799). In contrast, Aqualief^TM^ was found to be significantly effective in stimulating salivation, the flow rate after one week being 0.21 mL min^−1^, significantly higher compared to the mean value recorded at baseline (0.16 mL min^−1^; *p* = 0.0072) (Figure 1a). Figure 1b shows results as a percent increase between the two time points for each treatment. The relative variation of saliva flow rate in the placebo group was +0.03% ± 44.74 while in Aqualief^TM^ it was +30.48 ± 50.79. The difference between the two groups was statistically significant (*p* = 0.027). The graph in panel 1b shows also the distribution of the patients when divided into three groups: 1) no variation when the difference was between −10 and +10%, 2) increased salivation, when the difference was higher than 10% and 3) reduced salivation when lower than 10%. A higher percentage of patients in the Aqualief^TM^ group had an increase in saliva flow >10% compared to placebo (61 vs. 42%) while the percentage of subjects with reduced salivation was lower (21 vs. 42%). No difference was observed for the percentage of subjects reporting a difference between −10 and +10%, being 18 and 17% for Aqualief^TM^ and placebo, respectively. The difference between treatment groups (Aqualief^TM^ vs. placebo) was statistically significant when reporting the values as percent variation, but it was not significant for the absolute values (*p* = 0.6956) (Figure 1).

### 3.3. Secondary Endpoints

**Figure 2:**
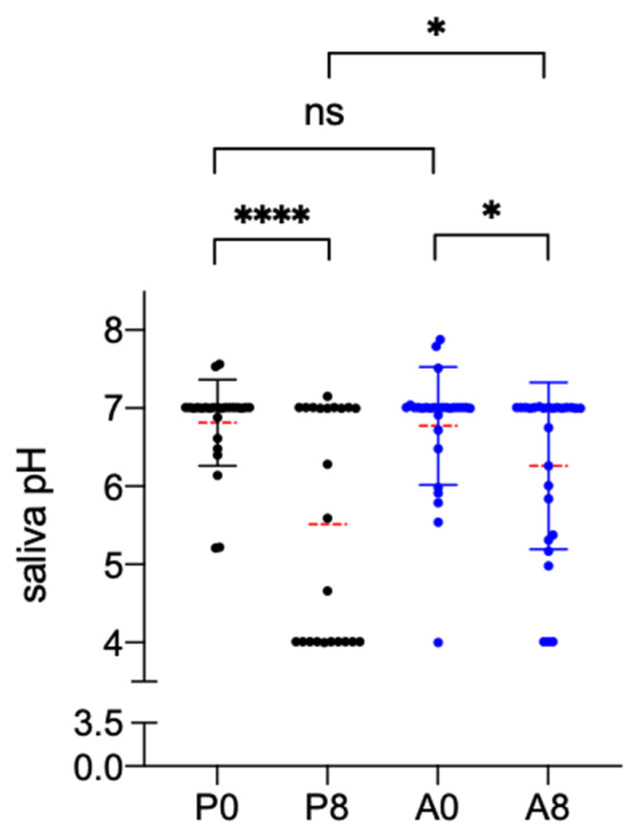
Effect of Placebo and Aqualief^TM^ treatment on saliva pH. Saliva pH, determined by potentiometric analysis, was recorded before (P0,A0) and after one week (P8,A8) of treatment. P is for placebo, A for Aqualief^TM^, * *p* < 0.1, **** *p* < 0.0001, ns, no significant differences. Statistical analysis between the two time points of the same group was performed by paired *t*-test, between the two groups by unpaired *t*-test.

After placebo treatment, the salivary pH between baseline and the subsequent visit had lowered by −1.30 (*p* = 0.0001) as measured with the pH meter. In particular, the pH value dropped from 6.81 ± 0.55 recorded at baseline to 5.51 ± 1.46 after 7 days. The pH reduction was also observed after Aqualief^TM^ treatment but to a much lesser extent: the mean value at the baseline was 6.77 ± 0.75 and 6.26 ± 1.07 after 7 days (−0.52, *p* = 0.0437). The difference between pH values recorded after 7 days of treatment between the two treatment groups was statistically significant (*p* = 0.0178) (Figure 2).

**Figure 3:**
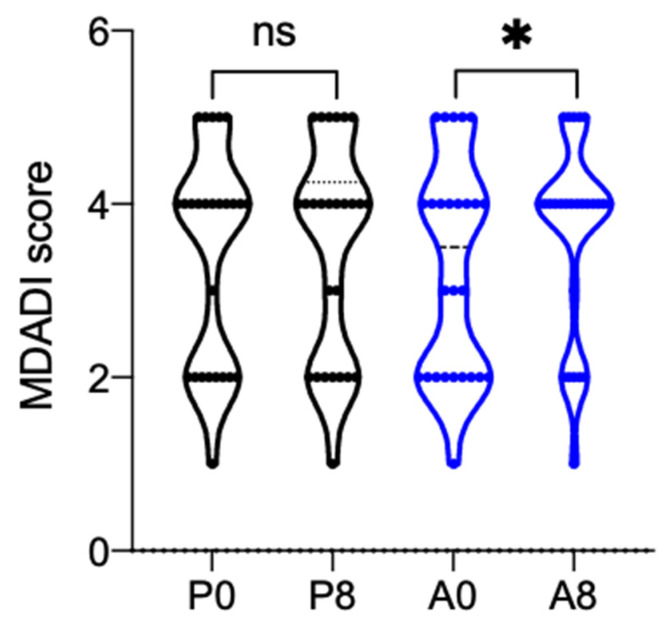
Violin plot of MDADI global score. MDADI global score recorded before (P0,A0) and after one week (P8,A8) of treatment. P is for placebo, A for Aqualief^TM^, * *p* < 0.1, ns, no significant differences. Statistical analysis between the two time points of the same group was performed by paired *t*-test, between the two groups by unpaired *t*-test.

As regards the questionnaires that were administered to the patients, a statistically significant increase in the MDADI Global Score was observed between baseline and the subsequent visit when Aqualief^TM^ was used (+0.38, *p* = 0.0221); in particular the mean value recorded at baseline was 3.27 ± 1.25 and increased to 3.65 ± 1.13 after one week of treatment. By contrast, no differences were observed in the placebo group (Figure 3). The MDADI Composite Score showed a different behavior. Here, the score increased when placebo was used (+1.30), while it decreased when Aqualief^TM^ was used (−1.05). The differences within each treatment group and between treatment groups, however, were not statistically significant. As regards the XQ-1 questionnaire, its score decreased more with placebo (−3.04) than with Aqualief^TM^ (−0.96). Again, however, the differences within each treatment and between treatments were not statistically significant.

The proportion of patients who were satisfied with the treatment received was slightly higher when Aqualief^TM^ was given (15.3 vs. 7.7%) (Table 3), but the difference in the distribution of answers between treatment groups was not statistically significant (*p* = 0.5702).

### 3.4. Adverse Events (AE)

Twelve AEs in 6 patients occurred when placebo was given, and 7 AEs occurred in 4 patients when Aqualief^TM^ was given. There was no statistically significant difference between the two groups. None of these AEs was serious and none of these needed a medication prescription for relief of symptom. Most were gastrointestinal disorders (aphthous ulcer, oral discomfort, dry mouth, etc.). One AE for each treatment group (i.e., placebo and Aqualief^TM^) and in particular the occurrence of aphthous ulcer led to premature termination of the study.

## 4. Discussion

This clinical study investigated the effects of a novel product, Aqualief^TM^, containing as its main ingredients carnosine and dried calyces of Hibiscus sabdariffa (karkadé), on the salivary function of patients treated with modern RT techniques for HNCs. Patients enrolled in this trial had a residual salivary capacity with RIX graded ≥2 according to CTCAE version 4.0 and unstimulated salivary flow between 0.1 and 0.25 mL/min. The study was stopped early because of poor accrual; however, some evidence was gained from this trial in favor of this novel treatment. The results show that after one week of therapy, the placebo was not effective in increasing salivation. In contrast, Aqualief^TM^ treatment was found to significantly increase salivation by almost 30% compared to baseline and almost 61% of the patients recorded an increase in salivation. When the data are reported as percent variation, hence calculating for each subject the difference between the two time points, a significant difference between Aqualief^TM^ and placebo was observed. However, when the results are analyzed as absolute values, no significance was found, and this is likely due to the relatively small number of patients that participated to this study.

Altogether these results are encouraging as regards the efficacy of Aqualief^TM^ in stimulating the salivation of HNC patients after RT. It is also encouraging that Aqualief^TM^ buffered the significant decrease of pH observed in placebo-treated patients. It should be underlined that the drop of pH observed in placebo-treated patients cannot be ascribed to the placebo tablets since they did not contain acid ingredients but is more likely due to the progressive pH reduction of the saliva typical of these patients. In fact, pH reduction of the saliva is one of the hallmarks of xerostomia and is of particular pathogenic relevance since it induces enamel and dental erosion and contributes to the creation of an inhospitable environment for protective bacteria favoring the outgrowth of aciduric bacteria [33].

The effect of Aqualief^TM^ on saliva pH can be explained by considering different mechanisms involving both carnosine and the karkadé extract, which probably act together. First, a direct buffering action of carnosine, which is an endogenous pH buffering agent, and it is effective in the skeletal muscle where it is present in mM concentrations [24]. Second, carnosine may modulate the saliva buffering activity by modulating the activity of carbonic anhydrase isoform VI (CA VI). CA VI is the only secretory isoenzyme of the mammalian CA family. It is exclusively expressed in the serous acinar cells of the parotid and submandibular glands, from where it is secreted into the saliva and incorporated into the protein pellicle on tooth enamel and in dental biofilms. CA VI supports neutralization of lactic and other bacteria-produced acids through conversion of saliva HCO_3_^−^ to water and carbon dioxide and pH maintenance through the subsequent phase buffering [34]. Carnosine was found to improve activation of four isoforms of human CA: I, II, V and IX [35] and we are currently investigating its effect on CA VI. Finally, the effect of Aqualief^TM^ on pH can also be ascribed to the increase in salivation as was found in this study. The karkadè extract can also modulate some effects due its well-established antioxidant effects and by its broad spectrum of antimicrobial activity due to different components including protocatechuic acid [36]. In any case, the above biological effects of the two constituents while on the one hand can explain the pH effect of Aqualief^TM^, on the other they do not resolve the salivation effect which for the moment remains an unexpected effect. Ongoing intervention studies based on quantitative proteomics are aimed at better understanding the mechanism of action of Aqualief^TM^ [37].

The effects of placebo and Aqualief^TM^ on salivation and pH agree well with the significant increase in the MDADI Global Score observed only for Aqualief^TM^.

Treatment of RIX has been addressed with several approaches which, however, have yielded, on a whole, unsatisfactory results so far. One approach rests on the use of salivary substitutes [21], in particular when the salivary gland damage is irreversible. Among the most commonly used products are sugarless chewing gum or xylitol/sorbitol candy or other substitutes [38,39,40]. These products are widely available without prescription, but they provide only short-lived relief and some studies have found that frequent sips of water are as effective [23]. Moreover, such substitutes lack some of the essential functions of the saliva, like antimicrobial and immunologic functions.

Given that stimulation of saliva production is certainly a more desirable approach but requires a residual function of salivary glands allowing saliva production to take place. In this class of products, pilocarpine, a muscarinic receptor agonist, has been extensively used. Systemic administration of pilocarpine has yielded mixed results as regards its efficacy and, in some studies, it was accompanied by side effects like sweating, headache, urinary frequency, vasodilatation, dizziness, dyspepsia, lacrimation and nausea [41,42,43]. Cevimeline is a parasympathomimetic/muscarinic agonist that has been investigated for the treatment of RIX in some studies [44,45] showing some improvement of xerostomia but at the expense of side effects, mostly sweating [41]. One of these trials, however, has questioned the efficacy of cevimeline in improving saliva flow and relieving xerostomia symptoms [45]. One clinical trial has compared pilocarpine and cevimeline [46] and found a slightly, but statistically not significantly, greater efficacy of pilocarpine in increasing saliva flow in patients with xerostomia. Other compounds that have been reported to be of some efficacy in the treatment of xerostomia with residual salivary gland function are anethol trithione, which simulates the parasympathetic nervous system, leading to increased secretion of acetylcholine and, consequently, to the stimulation of saliva secretion [47], and yohimbine, an α2-adrenergic receptor antagonist which leads, indirectly, to an increase of peripheral cholinergic activity [48]. Non-pharmacologic approaches that are used episodically for the treatment of xerostomia in these patients are electrical stimulation of the salivary glands [49] and acupuncture [50].

Finally, although the present study reported promising efficacy results in treating RIX with Aqualief^TM^ we must underline the following limitations due to unsuccessful trial accrual: limited safety data and little information about treatment effects in subgroups and QoL.

## 5. Conclusions

Aqualief^TM^ has shown positive results in patients with RIX, although with limitations due to unsuccessful trial accrual. Therefore, it may be further investigated as a tool for the treatment of this toxicity.

## Figures and Tables

**Table 1:** Demographics and Treatment Details of the Safety Population.

Characteristics		Placebo-Aqualief^TM^ (*N* = 14)	Aqualief^TM^-Placebo (*N* = 16)	*p*-Value
Gender	Males	9 (64.3%)	10 (62.5%)	0.919
	Females	5 (38.5%)	6 (37.5%)	
Age	N	14	16	
	Mean (SD)	58.07 (11.23)	58.56 (9.37)	0.897
	Median	58.50	57.50	
	Min–Max	39.00–74.00	40.00–75.00	
MDADI Global Score	N	14	16	0.502
	Mean (SD)	3.29 (1.07)	3.00 (1.21)	
	Median	4.00	2.00	
	Min–Max	2.00–5.00	2.00–5.00	
MDADI Composite Score	N	14	16	0.992
	Mean (SD)	70.15 (14.16)	70.20 (10.38)	
	Median	71.58	72.10	
	Min–Max	45.26–91.58	54.74–89.47	
XQ1-Questionnaire	N	14	16	0.830
	Mean (SD)	45.21 (16.04)	43.88 (17.51)	
	Median	43.00	46.00	
	Min–Max	19.00–72.00	19.00–73.00	

Abbreviations: MDADI, MD Anderson Dysphagia Inventory; XQ, xerostomia questionnaire.

**Table 2:** Demographics and Treatment Details of the modified ITT Population.

Characteristics		Placebo-Aqualief^TM^ (*N* = 13)	Aqualief^TM^-Placebo (*N* = 14)	*p*-Value
Gender	Females	8 (61.5%)	9 (64.3%)	0.883
	Males	5 (38.5%)	5 (35.7%)	
Age	N	13	14	
	Mean (SD)	58.62 (11.49)	58.00 (9.92)	0.883
	Median	59.00	55.50	
	Min–Max	39.00–74.00	40.00–75.00	
MDADI Global Score	N	13	14	0.614
	Mean (SD)	3.23 (1.09)	3.00 (1.24)	
	Median	4.00	2.00	
	Min–Max	2.00–5.00	2.00–5.00	
MDADI Composite Score	N	13	14	0.609
	Mean (SD)	68.99 (14.02)	71.35 (9.39)	
	Median	71.58	72.10	
	Min–Max	45.26–91.58	54.74–89.47	
XQ1-Questionnaire	N	13	14	0.871
	Mean (SD)	44.38 (16.38)	43.29 (18.31)	
	Median	39.00	46.00	
	Min–Max	19.00–72.00	19.00–73.00	

Abbreviations: ITT, intention to treat; MDADI, MD Anderson Dysphagia Inventory; XQ, xerostomia questionnaire.

**Table 3:** Summary and Analysis of Patients’ Global Satisfaction.

Answer	Placebo	Aqualief^TM^
Very poor (not satisfied at all)	5 (19.2%)	4 (15.4%)
Poor (not very satisfied)	9 (34.6%)	9 (34.6%)
Medium (on average satisfied)	10 (38.5%)	9 (34.6%)
Good (quite satisfied)	2 (7.7%)	3 (11.5%)
Very good (very satisfied)	0 (0.0%)	1 (3.8%)
*p*-value		0.572

## Data Availability

The datasets generated for this study are available on reasonable request to the corresponding author.
